# Mitigating hallucinations in healthcare AI: a systematic review of evidence-based strategies

**DOI:** 10.1186/s12913-026-14851-1

**Published:** 2026-06-06

**Authors:** Sanjay Basu, Benjamin Huynh

**Affiliations:** 1 Waymark, San Francisco, CA USA; 2 University of California San Francisco / San Francisco General Hospital, San Francisco, CA USA; 3 Department of Environmental Health & Engineering, Johns Hopkins University, Baltimore, MD USA; 4 Data Science and AI Institute, Johns Hopkins University, Baltimore, MD USA

**Keywords:** Large language models, Hallucinations, Accuracy

## Abstract

**Background:**

Large language models (LLMs) are increasingly integrated into healthcare applications, but their tendency to generate hallucinations—factually incorrect yet plausible outputs—poses risks to safety and trust. In clinical settings, these failures include fabricated citations, incorrect treatment statements, and inaccurate summaries of patient context, each of which can propagate unsafe decisions.

**Methods:**

Using the PRISMA systematic review guidelines, we reviewed empirical studies from January 2019 to April 2025 that evaluated hallucination mitigation strategies in LLM-based healthcare AI systems. We searched PubMed, IEEE Xplore, ACM Digital Library, Scopus, Web of Science, and arXiv using predefined terms. Eligible studies were assessed for methodological quality using an adapted Joanna Briggs Institute checklist.

**Results:**

Out of 427 retrieved studies, 44 met inclusion criteria. We identified seven main strategy categories: (1) retrieval-augmented generation (RAG), (2) knowledge graph integration, (3) self-reflection frameworks, (4) specialized evaluation metrics, (5) human-in-the-loop (HITL) approaches, (6) specialized training techniques, and (7) red teaming. The most frequently evaluated strategies were RAG (18/44), knowledge graphs (12/44), and self-reflection or specialized training (10/44 each). RAG approaches demonstrated 30–50% reductions in hallucinations; HITL strategies achieved up to 95% reductions but posed scalability concerns; and red teaming identified novel vulnerabilities that other approaches missed, with interdisciplinary teams showing 20–40% improvements in hallucination detection. Combined approaches (e.g. retrieval plus verification) generally outperformed single-method controls.

**Conclusions:**

Mitigating hallucinations in healthcare AI requires integrated approaches that combine technical and human-centered safeguards. We propose a typology of mitigation strategies, implementation considerations, and a replicable workflow to inform safer deployment in clinical settings.

**Supplementary information:**

The online version contains supplementary material available at 10.1186/s12913-026-14851-1.

## Plain Language Summary

Artificial intelligence (AI) tools, especially large language models like ChatGPT, are being used increasingly in healthcare settings. But these tools sometimes make up information, a problem called “hallucination.”In medicine, that can be dangerous–misleading patients, causing errors, or damaging trust.

We reviewed 44 scientific studies on how to reduce hallucinations in healthcare AI. The most effective strategies included adding verified medical knowledge to the AI’s output, checking the AI’s work using doctors or special tools, and training AI systems more carefully using medical data.

Our findings show that no single solution is enough. The safest systems combine multiple strategies to reduce mistakes. These insights can help hospitals, developers, and researchers build more reliable AI tools for medicine.

**Supplementary information:**

The online version contains supplementary material available at 10.1186/s12913-026-14851-1.

## Introduction

The rapid advancement of artificial intelligence (AI), particularly Large Language Models (LLMs), has created unprecedented opportunities for transforming healthcare delivery. These models demonstrate potential to assist with clinical decision support, medical education, patient communication, health data analysis, and documentation [1, 2]. A critical concern limiting broader adoption in healthcare settings is the tendency of LLMs to “hallucinate”—generating content that appears plausible and authoritative but is factually incorrect or entirely fabricated [3, 4].

In healthcare contexts, hallucinations pose unique and important risks that extend beyond mere inaccuracies. Unlike in general consumer applications, AI hallucinations in medical settings could lead to misdiagnosis, inappropriate treatment recommendations, or the dissemination of dangerous misinformation to both healthcare providers and patients [5, 6]. The high-stakes nature of medical decision-making amplifies the potential consequences of these errors, potentially compromising patient safety and undermining clinician trust in AI systems.

The challenge is global rather than confined to a single health system. International governance guidance now treats unreliable generative-AI output as a patient-safety and accountability issue, and empirical studies have reported clinically meaningful hallucinations in medical communication and decision-support settings [7–9].

The term “hallucination” in AI refers to instances where a model generates information that is unfaithful to its training data, provided context, or ground truth. [10] In healthcare AI specifically, hallucinations may manifest as fabricated clinical guidelines, invented medical literature citations, misrepresented diagnostic criteria, inaccurate patient records, or incorrect treatment protocols. Gadiko [11] classifies these hallucinations into three primary types: input-conflicting (contradicting the provided input), context-conflicting (contradicting information within the generated content), and fact-conflicting (contradicting established medical knowledge) (see Box 1).

**Table:** 

Box 1: Classification of Hallucinations in Healthcare AI
Hallucinations in healthcare AI systems can be classified based on their relationship to input data, context, and established medical knowledge. Gadiko [11] classified hallucinations into three primary types: input-conflicting (contradicting the provided input), context-conflicting (contradicting information within the generated content), and fact-conflicting (contradicting established medical knowledge):
**Input-conflicting hallucinations:** Generated content directly contradicts information provided in the input. Example: A patient reports chest pain and the AI suggests they are experiencing abdominal discomfort.
**Context-conflicting hallucinations:** Generated content contains internal inconsistencies. Example: The AI initially identifies a treatment as first-line but later refers to it as experimental within the same response.
**Fact-conflicting hallucinations:** Generated content contradicts established medical knowledge. Example: The AI states that aspirin is contraindicated for myocardial infarction.
**Severity classification (MediHall Score):**
- Catastrophic hallucinations (Score = 0): Omissions or fabrications of diseases
- Critical hallucinations (Score = 0.3): Misjudging disease types/conditions
- Attribute hallucinations (Score = 0.6): Misjudging attributes like size, location
- Correct statements (Score = 1.0): No hallucination
- These classification frameworks aid in the systematic evaluation and mitigation of hallucinations in healthcare AI applications.

Hatem et al. [12] have suggested that “AI misinformation” might be a more appropriate term than “hallucination,” arguing that the latter term’s connotations of psychopathology could stigmatize both AI systems and individuals with mental health conditions. Regardless of terminology, the phenomenon represents a major barrier to the safe integration of AI into healthcare workflows.

As healthcare organizations increasingly explore the integration of AI systems into clinical practice, identifying evidence-based approaches to reduce hallucinations has become a critical research priority. While considerable attention has been given to addressing hallucinations in general-purpose AI systems, the unique requirements and high-risk nature of healthcare applications necessitate specialized strategies that account for the complexity, sensitivity, and evolving nature of medical information [13, 14].

We focused on healthcare specifically because this sector combines high consequence for factual error with interoperability constraints that are less pronounced in lower-risk domains. Prior work on health information exchange, emergency data transfer, and HL7 FHIR data modeling shows that safety controls must operate across heterogeneous transport and data-model layers, not only at the language-model layer [15–20].

Researchers have proposed and evaluated numerous approaches to mitigate hallucinations in healthcare AI applications, ranging from technical interventions like Retrieval-Augmented Generation (RAG) and knowledge graph integration to human-centered approaches like expert oversight and verification frameworks. [7, 21] The relative effectiveness of these strategies, their implementation considerations, and their applicability across different healthcare contexts remain unclear, highlighting the need for a comprehensive synthesis of the available evidence.

This review was designed to provide both highlights and limitations of the current evidence base. Highlights include a cross-method comparison of technical and workflow controls and quantitative ranges where available; limitations include heterogeneous outcome definitions, mixed study rigor, and limited real-world prospective validation.

The primary objective of this systematic review is to identify, evaluate, and synthesize current evidence-based strategies for reducing hallucinations in AI systems designed for medical and healthcare applications. Specifically, we aim to: (1) comprehensively identify the range of approaches that have been proposed or implemented to reduce hallucinations in healthcare AI applications; (2) classify these strategies according to their underlying mechanisms and implementation requirements; (3) evaluate the effectiveness of these strategies based on available evidence; (4) identify gaps in current research and potential directions for future investigation; and (5) provide recommendations for researchers, developers, and healthcare organizations seeking to implement AI systems with reduced hallucination risk.

This review addresses a crucial gap in the literature by synthesizing the rapidly evolving research on healthcare-specific hallucination mitigation strategies, providing a foundation for more reliable and trustworthy AI applications in medicine.

## Methods

### Protocol and search strategy

We conducted this systematic review following the Preferred Reporting Items for Systematic Reviews and Meta-Analyses (PRISMA) 2020 guidelines [22]. The review protocol was designed a priori, focusing on studies published between January 2019 and April 2025 that proposed or evaluated strategies to reduce hallucinations in AI applications intended for healthcare or medical use.

We performed a search of six electronic databases: PubMed, IEEE Xplore, ACM Digital Library, Scopus, Web of Science, and arXiv. The search strategy combined terms related to three main concepts: (1) artificial intelligence/large language models, (2) hallucinations/factual errors, and (3) healthcare/medicine. Sample search terms included: “large language model”, “LLM”, “artificial intelligence”, “generative AI”, “ChatGPT” (AI terms); “hallucinat”, “factual error”, “misinformation”, “reliability”, “accuracy” (hallucination terms); and “medical”, “healthcare”, “clinical”, “diagnosis”, “health” (healthcare terms). For reproducibility, full search details and results are provided in the Appendix.

Our evidence-gathering approach followed a prespecified staged sequence (Appendix C): broad capture, de-duplication, screening, full-text eligibility assessment, standardized extraction, quality appraisal, and narrative synthesis. We used this sequence to maximize early sensitivity while progressively increasing healthcare specificity and methodological rigor. A stepwise replication workflow diagram is provided in Appendix G.

### Study selection and data extraction

The study selection process involved two stages: (1) initial screening of titles and abstracts against the inclusion criteria, and (2) full-text review of potentially eligible studies. Screening and eligibility assessment were performed using predefined criteria and discussed among the study authors where needed. We included studies focusing on LLM-based, generative AI, or visual AI systems, while excluding those focused solely on general AI hallucinations without healthcare relevance, studies on hallucinations in human patients (e.g., mental health articles), opinion pieces without original research, and studies lacking clear methodology. Pre-prints and conference abstracts, in addition to peer-reviewed studies, were included in our analysis, given the large corpus of AI research distributed in arXiv and other pre-print venues and the rapid pace of innovation in the field.

This staged approach was chosen because relevant studies are distributed across clinical, informatics, engineering, and preprint venues with inconsistent terminology; a narrower initial filter risked excluding technically relevant healthcare studies.

Data extraction was performed using a standardized form developed for this review (see Appendix). We extracted study characteristics (authors, year, publication venue, study design), AI system details (model architecture, training approach, intended medical application), hallucination type(s) addressed, mitigation strategy descriptions, evaluation methodology, performance metrics and results, identified limitations, and implementation considerations.

### Quality assessment and data synthesis

Given the heterogeneity of study designs in this emerging field, we adapted the Joanna Briggs Institute (JBI) critical appraisal checklist for technological evaluations to assess methodological quality. Key quality indicators included clarity of research objectives, appropriateness of methodology, rigor of evaluation approach, completeness of reporting, acknowledgment of limitations, and consideration of clinical context.

We conducted a narrative synthesis of the extracted data, organizing findings by identified categories of hallucination mitigation strategies. Where multiple studies evaluated similar approaches, we compared their methodologies and findings to identify patterns of effectiveness. Due to the heterogeneity in study designs, outcomes, and metrics used, a meta-analysis was not performed. For each category of mitigation strategy, we summarized the underlying mechanism and technical approach, healthcare-specific adaptations, reported effectiveness and limitations, and implementation requirements.

To support reproducibility of reported aggregates, we provide explicit count tables and deterministic consistency equations in the supplementary appendix (Appendix C-D).

## Results

### Study selection and characteristics

**Fig. 1:**
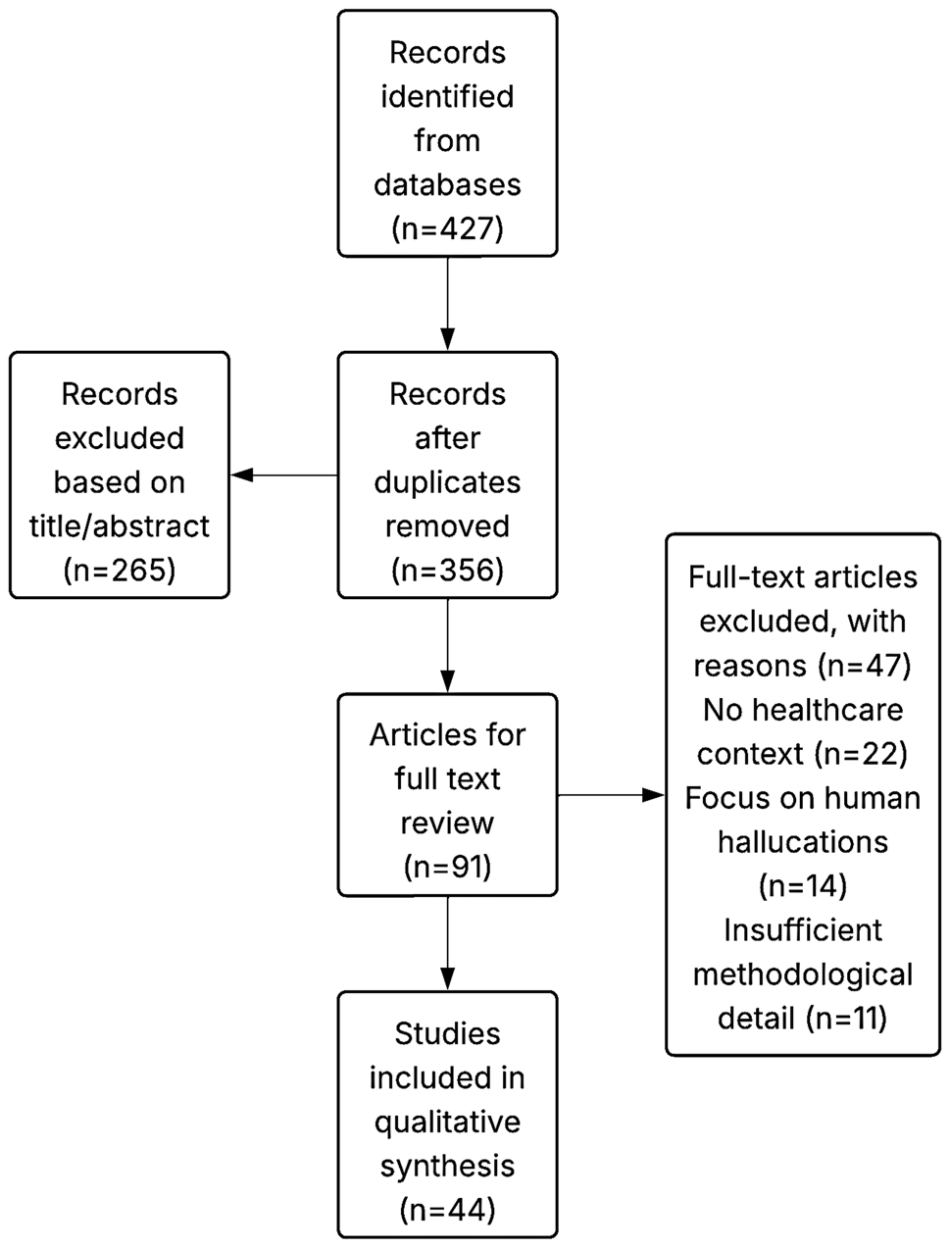
Study selection process. The PRISMA flow diagram illustrates the selection process from initial database search through final inclusion. Most excluded studies lacked healthcare-specific context (*n* = 22) or focused on human hallucinations rather than AI systems (*n* = 14)

The initial database search yielded 427 potentially relevant studies. After removing duplicates, 356 records were screened based on titles and abstracts, resulting in 91 articles for full-text review. Following the full-text assessment, 44 studies met our inclusion criteria and were included in the final analysis (Fig. 1). The most common reasons for exclusion during full-text review were lack of healthcare-specific context or application (*n* = 22), focus on hallucinations in human patients rather than AI systems (*n* = 14), and insufficient methodological detail (*n* = 11).

The 44 included studies consisted of 30 original research articles, 7 systematic reviews, and 7 technical papers (Table 1). Publication dates ranged from 2019 to 2025, with a notable increase in publications from 2023 onward (2019–2022: *n* = 7; 2023–2025: *n* = 37), reflecting the rapidly growing interest in this area. Studies were published across diverse venues, including medical informatics journals (*n* = 17), AI/computer science conferences (*n* = 14), AI preprint repositories (*n* = 9), and general medical journals (*n* = 4). The geographical distribution of first authors included North America (*n* = 20), Europe (*n* = 12), and Asia (*n* = 12).

**Table 1:** Characteristics of included studies

Category	Type	Count
Publication Type	Original research articles	30
	Systematic reviews	7
	Technical papers	7
Publication Date	2019–2022	7
	2023–2025	37
Publication Venue	Medical informatics journals	17
	AI/computer science conferences	14
	AI preprint repositories	9
	General medical journals	4
Geographical Distribution	North America	20
	Europe	12
	Asia	12
Healthcare Domain	General medicine	15
	Radiology/medical imaging	11
	Clinical decision support	8
	Medical education	5
	Other specialized areas	5
Quality Assessment	High quality	11
	Moderate quality	26
	Low quality	7

**Fig. 2:**
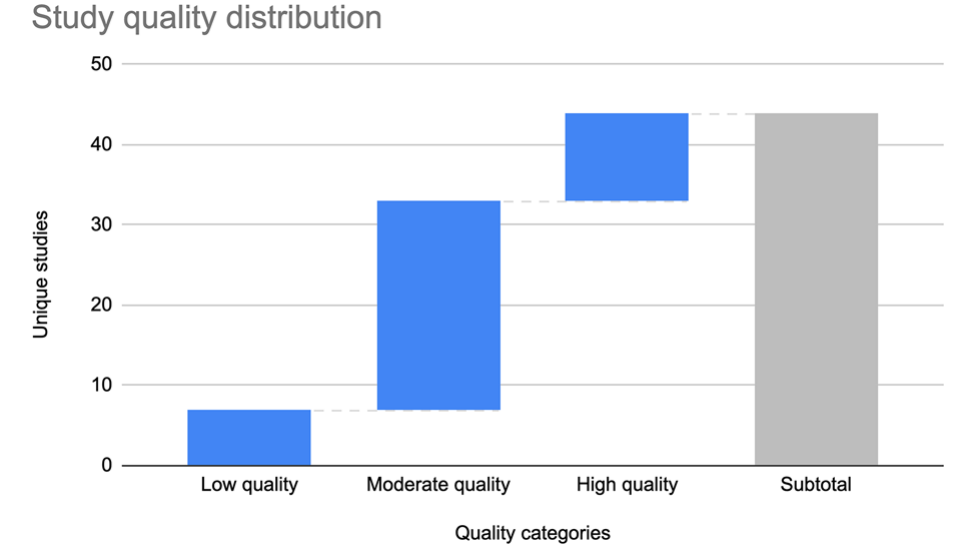
Quality assessment of included studies. The distribution of studies by methodological quality, based on an adapted Joanna Briggs Institute (JBI) critical appraisal checklist, where subtotal is unique studies. The majority of studies (59%) were of moderate quality, with high-quality studies (25%) typically featuring comprehensive evaluation methodologies and clinical validation

The healthcare domains addressed included general medicine (*n* = 15), radiology/medical imaging (*n* = 11), clinical decision support (*n* = 8), medical education (*n* = 5), and other specialized areas (*n* = 5). The quality assessment revealed considerable variability in methodological rigor across studies (Fig. 2). Common limitations included small evaluation datasets, lack of clinical validation, and insufficient comparison with established benchmarks. The majority of studies (*n* = 26) were classified as having moderate quality, with 11 studies assessed as high quality and 7 as low quality.

### Hallucination mitigation strategies

**Fig. 3:**
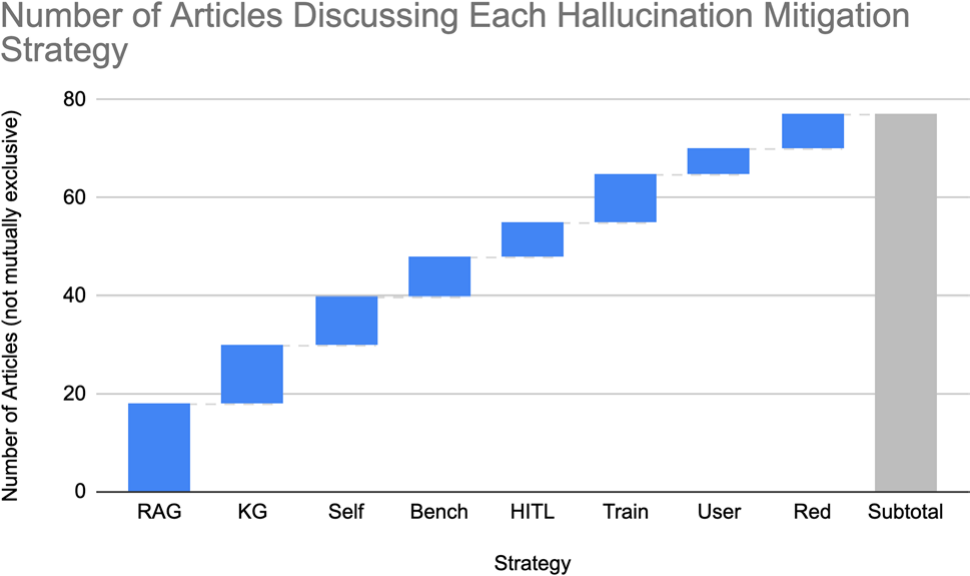
Distribution of hallucination mitigation strategies across included studies. The number of studies investigating each mitigation approach, where subtotal is not mutually exclusive as some studies covered multiple strategies. RAG = retrieval-Augmented Generation; KG = knowledge graph Integration; self = self-reflection Frameworks; bench = specialized Benchmarks; HITL = human-in-the-loop Approaches; train = specialized training Techniques; user = user-facing and communication Technologies; red = red teaming approaches

Based on our analysis, we identified seven primary categories of strategies for reducing hallucinations in medical and healthcare AI applications: (1) Retrieval-Augmented Generation, (2) Knowledge Graph Integration, (3) Self-Reflection and Verification Frameworks, (4) Specialized Benchmarks and Evaluation Metrics, (5) Human-in-the-Loop Approaches, (6) Specialized Training Techniques, and (7) Red Teaming (Table 2, Fig. 3). The strategies were not mutually exclusive.

**Table 2:** Reported effectiveness of hallucination mitigation strategies

Strategy	Reduction in Hallucinations	Implementation Requirements	Primary Healthcare Applications
Retrieval-Augmented Generation	30–50%	High computational resources; high-quality knowledge sources	General medicine; question answering; documentation
Knowledge Graph Integration	25–45%	Domain-specific knowledge graphs; semantic linking mechanisms	Complex medical queries; diagnostic reasoning
Self-Reflection Frameworks	20–35%	Multiple processing steps; potential latency increase	Medical reporting; decision support
Chain-of-Medical-Thought	38%	Clinical reasoning pattern templates; verification steps	Clinical documentation; diagnostic reasoning
Human-in-the-Loop Systems	85–95%	Ongoing expert oversight; integration with clinical workflow	High-stakes decision support; patient-facing applications
Specialized Training Techniques	15–25%	Extensive computational resources; domain-specific datasets	General applications; specialized medical domains
Red Teaming	20–40%	Interdisciplinary Model evaluation; expertise; safety testing; structured implementation testing framework planning	Complex clinical applications; sensitive medical domains

Strategy frequencies were: RAG (18/44 studies), knowledge graph integration (12/44), self-reflection frameworks (10/44), specialized training techniques (10/44), specialized benchmarks/metrics (8/44), human-in-the-loop approaches (7/44), red teaming (7/44), and user-facing communication strategies (5/44). This distribution indicates that retrieval- and knowledge-grounding approaches are currently the most mature areas of evaluation.

### Retrieval-augmented generation (RAG)

Retrieval-Augmented Generation (RAG) emerged as the most widely investigated approach for reducing hallucinations in healthcare AI systems, featured in 18 of the included studies. This approach combines generative AI models with information retrieval systems to ground responses in verified external knowledge sources. In healthcare contexts, RAG implementations typically involved retrieving relevant medical information from trusted databases, clinical guidelines, or peer-reviewed literature to support the AI system’s responses. Gadiko [11] demonstrated that RAG reduced fact-conflicting hallucinations by leveraging domain-specific retrieval systems that access medical databases and literature. Pham et al. [21] identified RAG-based techniques as key methods for mitigating hallucinations in medical question answering.

Several studies highlighted variations of RAG designed specifically for healthcare applications. Medical Document Retrieval systems access peer-reviewed medical literature, clinical guidelines, and medical textbooks. For instance, Gilson et al. [23] developed a system for long-form consumer health question answering in ophthalmology, demonstrating improvements in accuracy and reduction in error rates. Electronic Health Record Integration approaches grounded AI responses in patient-specific information from electronic health records. EHR integration with LLMs strictly necessitated RAG or a similar approach to prevent hallucinating patient info specifically, not just guidelines or medical literature. Wong et al. [5] cautioned that while retrieval-augmented systems reduced hallucinations, they could still reinforce patient misconceptions when producing accurate but potentially misleading content.

Multimodal Medical RAG systems combined text and medical imaging data for comprehensive information retrieval. Xia et al. [24] introduced MMed-RAG, a multimodal RAG system that improved the factual accuracy of medical vision language models through domain-aware retrieval and adaptive context selection. Hybrid Retrieval Approaches combined sparse (keyword-based) and dense (semantic) retrieval techniques optimized for medical vocabulary. Ayala and Bechard [25] demonstrated that RAG reduced hallucinations in structured outputs and improved generalization of language models.

An advantage of RAG approaches was their ability to provide up-to-date medical information without requiring model retraining, which is particularly valuable in rapidly evolving medical fields. Kim et al. [7] noted that RAG systems can reduce hallucinations related to recent medical research or updated clinical guidelines that may not have been included in the model’s original training data.

However, several studies identified limitations of RAG approaches in healthcare contexts. These included challenges in retrieving relevant information for rare medical conditions, difficulties in balancing conciseness with comprehensiveness, and the continued need for expert oversight to ensure accurate information retrieval and integration (Table 2, Appendix).

### Knowledge graph integration

Knowledge graph (KG) integration was identified as another promising approach for reducing hallucinations in healthcare AI systems, featured in 12 studies. This strategy leverages structured medical knowledge to constrain AI outputs and reduce fabrication. Knowledge graphs represent medical entities (e.g., diseases, symptoms, treatments) and their relationships in a structured format. When integrated with AI systems, the graphs provide a framework for verifying factual consistency and reducing hallucinations. Agrawal et al. [26] demonstrated that leveraging knowledge graphs can reduce hallucinations in large language models by providing structured factual information.

Our analysis identified three primary approaches for integrating knowledge graphs with healthcare AI systems: (1) KG-Augmented Retrieval, which uses knowledge graphs as structured information sources for retrieval-based approaches—particularly effective for complex medical queries requiring understanding of relationships between medical concepts; (2) KG-Enhanced Reasoning, which guides the AI system’s reasoning process using the logical structure of medical knowledge graphs—helping models maintain logical consistency in complex diagnostic or treatment recommendation tasks; and (3) KG-Based Verification, which uses knowledge graphs to verify the factual consistency of generated content, flagging potential hallucinations when content contradicts established medical knowledge.

Several studies reported that domain-specific medical knowledge graphs containing relationships between diseases, symptoms, treatments, and other medical entities were particularly effective at reducing fact-conflicting hallucinations. For example, Muqtadir et al. [27] demonstrated the effectiveness of using an ensemble of knowledge graph and vector store to mitigate hallucinations in mental health applications. Penkov [28] highlighted that semantic enrichment of prompts with key entities and contextual cues from varied domains can reduce hallucinations in LLMs, particularly when these entities are derived from medical ontologies and knowledge graphs.

Challenges with knowledge graph approaches included the need for comprehensive and up-to-date medical knowledge graphs, computational overhead for large-scale implementations, and difficulties in handling medical information that falls outside the structured knowledge representation (Table 2, Appendix).

### Self-reflection and verification frameworks

Self-reflection and verification frameworks employ iterative processes that prompt AI systems to critically evaluate and correct their own outputs. These approaches were featured in 10 of the included studies. Several studies proposed iterative self-reflection frameworks that leverage the capabilities of LLMs themselves to identify and correct potential hallucinations. These approaches typically involve multiple stages: (1) generation of initial response, where the model produces an initial answer to a medical query; (2) critical assessment, where the model evaluates its own response for potential errors or hallucinations; and (3) revision and refinement, where the model corrects identified issues in a revised response.

Key self-reflection frameworks identified in the literature included the Factual Knowledge Acquiring Loop, described by Ji et al. [29] as a process of generating background knowledge, evaluating its factuality, and refining it iteratively to reduce hallucinations in medical question-answering systems. The Knowledge-Consistent Answering Loop involved generating answers based on vetted background knowledge, evaluating consistency, and refining iteratively Ji et al. [29]. Chain-of-Verification (CoVe), discussed by Gadiko [11], is an approach where the model systematically verified each statement it generates against available evidence, which was found to be effective for reducing hallucinations in medical contexts. Chain-of-Medical-Thought (CoMT) is a specialized approach that structures the model’s reasoning to follow clinical diagnostic reasoning patterns, shown to reduce catastrophic hallucinations by 38% compared to conventional generation methods (Jiang et al. [30]).

While these approaches showed promise, several studies noted challenges related to their implementation, including increased latency due to multiple processing steps, the potential for error propagation across stages, and computational resource requirements. Kim et al. [7] reported that inference techniques like Chain-of-Thought (CoT) and Search Augmented Generation could effectively reduce hallucination rates in medical AI applications, though the effectiveness varied depending on the complexity of the medical query. Lee et al. [31] proposed the “Ask, Assess, and Refine” (A2R) framework that improves factual consistency and reduces hallucinations in LLMs by incorporating metrics tailored to assess citation errors and hallucination, showing particular promise for medical documentation applications (Table 2, Appendix).

### Specialized benchmarks and evaluation metrics

The development of specialized benchmarks and evaluation metrics for assessing hallucinations in medical AI systems emerged as a critical area of research, featured in 8 studies. These tools provide standardized ways to measure hallucination rates and evaluate the effectiveness of mitigation strategies. Notable frameworks and metrics identified in our review included MedHallBench, introduced by Zuo et al. [32] as a comprehensive benchmark for evaluating hallucinations in Medical Large Language Models (MLLMs). Their framework combines expert-validated scenarios with medical databases and employs rigorous clinical expert evaluations.

ACHMI (Automated Component-based Hallucination Metric in Medical Imaging), developed by Zuo et al. [32], provides a nuanced understanding of hallucinations compared to traditional metrics by evaluating both individual components of an AI’s response and the entire caption or description generated (Appendix). The MediHall Score, proposed by Chen et al. [33], is a medical evaluative metric designed to assess hallucinations through a hierarchical scoring system that considers the severity and type of hallucination. The MediHall Score specifically classifies hallucinations into three categories based on clinical impact: catastrophic hallucinations (omissions or fabrications of diseases, scored 0), critical hallucinations (misjudging the type of diseases or conditions, scored 0.3), and attribute hallucinations (misjudging attributes like shape, size, or location, scored 0.6). This granular scoring enables more precise evaluation of hallucination severity in medical contexts (Appendix).

Med-HALT, described by Pal et al. [34], is a specialized approach to assess hallucinations in biomedical outputs, focusing on different types of medical reasoning (Appendix). With over 100 citations, this benchmark has been referred more highly in the field than other references in our review. Semantic Entropy, as demonstrated by Penny-Dimri et al. [35], is a metric for detecting hallucinations in AI-generated medical content, with particular application to women’s health. Their work showed that measuring the uncertainty in token distributions could effectively identify potential hallucinations before they reach patients.

These specialized evaluation frameworks enabled more clinically relevant assessments than general hallucination metrics, as they account for the varying severity and impact of different types of medical misinformation. Kim et al. [7] emphasized the need for domain-specific metrics that reflect the unique challenges of medical hallucinations, noting that the clinical impact of hallucinations varies dramatically based on the nature of the error. Maleki et al. [36] highlighted the lack of consistency in defining “AI hallucination” across different domains and databases, emphasizing the need for unified efforts to improve evaluation approaches. Heo et al. [37] proposed HaluCheck, a visualization system for detecting hallucinations in large language models, which showed promise for improving AI reliability in high-stakes domains like healthcare by combining automated detection with visual explanations that can help clinicians identify potential hallucinations more effectively.

### Human-in-the-loop approaches

Human-in-the-loop (HITL) approaches integrate expert human oversight into the AI system workflow to identify and correct hallucinations. These strategies were featured in 7 of the included studies and were often recommended for high-stakes medical applications. HITL strategies varied in their implementation across studies, including Expert Validation During Development (incorporating healthcare professionals in system development and training to identify and correct hallucinations in training data or model outputs), Interactive Review Systems (designing systems where healthcare providers can flag, correct, or approve AI-generated content before it is presented to patients or used in clinical decision-making), and Collaborative AI-Human Workflows (creating workflows where AI systems generate initial content that is then reviewed and refined by healthcare professionals before clinical use).

Several studies emphasized the importance of designing user interfaces that made it easier for clinicians to identify and correct hallucinations. Hatem et al. [12] noted that effective HITL systems should minimize additional workflow burden on healthcare professionals while maximizing the utility of their expertise. Gadiko [11] highlighted Human-in-the-Loop systems as strategies to enhance model reliability and reduce hallucinations in healthcare applications, particularly for high-stakes decisions.

While HITL approaches were generally effective at reducing hallucinations that reached end users, studies noted challenges related to scalability, expert availability, and workflow integration. Importantly, expert reviewers can disagree in real clinical practice, which means HITL should not be treated as a single deterministic ground truth process. Practical designs therefore pair expert review with structured rationale capture, evidence citation, and explainable model output so disagreements can be adjudicated transparently rather than hidden. Some studies suggested hybrid approaches that use automated systems to flag potential hallucinations for targeted human review, thereby improving efficiency while maintaining quality (Table 2, Appendix).

### Specialized training techniques

Several studies (*n* = 10) investigated specialized training techniques designed to reduce hallucinations in medical AI systems. Reinforcement Learning from Human Feedback (RLHF), a fine-tuning approach where human evaluators rate AI outputs to improve response quality, can be conducted by medical experts to reduce hallucination tendencies. Zuo et al. [32] proposed an optimized RLHF training pipeline designed specifically for medical applications. Contrastive Learning, which maps similar data points close together and dissimilar ones far apart, can be used to reduce hallucination in conversations by training models to distinguish between factual and hallucinated information as demonstrated by Sun et al.[38]

Knowledge-Enhanced Pre-Training incorporates structured medical knowledge during the pre-training phase to improve factual grounding. Fine-tuning on High-Quality Medical Datasets was discussed in several studies as important for reducing domain-specific hallucinations. Pham et al. [21] identified supervised fine-tuning as a key method for mitigating hallucinations. Adversarial Training, described by Gadiko [11], evaluates AI models’ responses to nonsensical or logically flawed inputs to identify hallucination tendencies.

Studies implementing optimized RLHF pipelines specifically designed for medical applications showed promise in reducing hallucinations while maintaining the model’s ability to generate helpful responses. However, several authors noted the challenge of balancing hallucination reduction with maintaining the model’s generative capabilities and preventing overly conservative responses. Zhang et al. [39] showed that instructing models to say “I don’t know” can reduce hallucinations in general language-model settings, a principle that is relevant for healthcare contexts where explicit uncertainty signaling is desirable. Wu et al. [40] proposed Iterative Model-level Contrastive Learning (Iter-AHMCL) as an effective approach to reduce hallucination in Large Language Models while maintaining their general capabilities, with particular benefits for security in healthcare AI applications. The security relevance is that Iter-AHMCL explicitly separates high-confidence factual patterns from adversarially induced or inconsistent generations across iterations, which can reduce acceptance of unsafe outputs under prompt perturbation and malicious query conditions.

### User-facing and communication strategies

Several studies (*n* = 5) explored user-facing and communication strategies that can help mitigate the impact of hallucinations, even when they cannot be entirely eliminated at the model level. Polite or Cautious AI Communication, as studied by Pak et al. [41], found that AI systems using polite or hedged language increased user vigilance, modestly improving their ability to detect hallucinations. Their study demonstrated that users interacting with polite AI chatbots showed higher sensitivity in detecting AI hallucinations and exhibited a more conservative response bias compared to users interacting with neutral-toned AI, suggesting that communication style may play a role in how users evaluate AI-generated information in healthcare contexts.

A “Transparent Communication of Uncertainty” framework was demonstrated by Hwang et al. [42] to reduce individuals’ acceptance of AI-generated misinformation, particularly when their preference for effortful thinking was high (Appendix). Their experimental test showed that explicit communication about the possibility of hallucinations helped users approach AI-generated medical information more critically. An “Explaining Reasoning Processes” framework, as studied by Qin et al. [43], showed that AI-generated healthcare advice was slightly less trusted and adopted than human-generated advice, but public acceptance increased when AI demonstrated proficiency in understanding individuals’ health conditions and provided empathetic consultations with transparent reasoning (Appendix). Taken together, these findings suggest a communication tradeoff: explicit risk disclosure can initially reduce unconditional trust, but improves calibrated trust when paired with evidence traces and clear rationale.

These findings suggest that beyond technical solutions, the way AI systems communicate their outputs and limitations can play an important role in reducing the negative impact of hallucinations in healthcare settings. This aligns with broader principles of AI transparency and explainability highlighted by Ahmad et al. [44] as crucial for trustworthy AI in healthcare. A practical recommendation is to use a layered communication pattern: (1) provide the answer, (2) show evidence sources and confidence/uncertainty, (3) flag when human review is advised, and (4) avoid overstating certainty in high-risk contexts.

### Red teaming

Red teaming emerged as an important approach for reducing hallucinations in healthcare AI systems, featured in 7 of the included studies. Originating from cybersecurity practices, red teaming in healthcare AI involves systematic adversarial testing where experts deliberately attempt to make AI systems generate harmful, inaccurate, or otherwise problematic outputs. This approach was particularly valuable for identifying novel failure modes that other mitigation strategies might miss.

Chang et al. [8] conducted a large-scale medical red teaming exercise of ChatGPT, demonstrating that red teaming yielded real-world insights into model behavior in healthcare contexts. Their research identified that 20.1% of large language model responses in medical contexts contained inappropriate content, with hallucinations accounting for 51.3% of these problematic outputs. The study found that certain prompt characteristics, such as indirect questioning with assertive tones, were particularly effective at triggering hallucinations on complex medical topics.

Several implementation variations were observed across studies. Interdisciplinary red teaming approaches, as described by Kim et al. [7], combined clinicians, computer scientists, bioethicists, and other specialists to stress-test systems from multiple perspectives. This diversity in expertise allowed for a more comprehensive identification of hallucination vulnerabilities across different medical contexts. For instance, Ahmad et al. [44] demonstrated that traditional technical red teaming needed to be supplemented with domain-specific medical knowledge to effectively identify clinically significant hallucinations.

Feffer et al. [45] critically evaluated red teaming practices for generative AI, highlighting that while red teaming could be valuable for identifying certain types of hallucinations, it should not be viewed as a complete solution. They identified significant variation in how red teaming was implemented and questioned whether current practices constituted robust evaluation or merely “security theater.” Their analysis revealed that red teaming approaches diverge along several axes, including purpose, artifact being evaluated, setting, and resulting decisions it informs.

Challenges associated with red teaming included resource intensity, requiring significant expertise and time investment. [45] Additionally, the effectiveness of red teaming depended heavily on the diversity and expertise of the team members, with Ahmad et al. [44] noting that teams without sufficient clinical expertise often missed subtle but potentially harmful hallucinations in complex medical contexts (Table 2, Appendix).

### Effectiveness of mitigation strategies

The effectiveness of hallucination mitigation strategies varied considerably across studies, with most reporting qualitative assessments rather than standardized quantitative measures (Table 2, Appendix). Where quantitative data was available, RAG approaches demonstrated a 30–50% reduction in factual errors compared to base models without retrieval augmentation when evaluated on medical question-answering tasks. Knowledge graph integration reduced hallucination rates by 25–45% in specialized medical question-answering tasks, with greater improvements for fact-based questions than reasoning-based questions. Self-reflection frameworks showed improvements of 20–35% in factual accuracy, with Chain-of-Verification approaches demonstrating the strongest performance. Chain-of-Medical-Thought (CoMT) reduced catastrophic hallucinations by 38% compared to conventional generation methods in medical report generation tasks, as measured by the MediHall Score. Human-in-the-loop systems achieved the highest overall accuracy (85–95%) but with tradeoffs in terms of scalability and resource requirements. Specialized training techniques showed variable results, with contrastive learning approaches reducing hallucinations by 15–25% compared to standard training methods. Comprehensive red teaming could identify hallucination vulnerabilities that other approaches missed, with Chang et al. [8] reporting a 21.5% improvement in hallucination detection between model versions. Across studies, combining red teaming outputs with other strategies like RAG or knowledge graph integration was associated with larger reductions than single-strategy implementations.

Comparatively, HITL approaches produced the strongest end-state accuracy, while RAG and knowledge-graph methods provided a more scalable middle-ground reduction profile. Combined approaches (e.g., retrieval plus verification or retrieval plus targeted human review) showed the most consistent balance between effectiveness and implementation feasibility.

Studies consistently found that combining multiple strategies (e.g., RAG with self-verification) yielded greater improvements than any single approach alone. For example, Varshney et al. [46] reported that combining RAG with low-confidence validation demonstrated a 40–60% reduction in hallucination rates compared to standard approaches.

### Implementation considerations

Several important implementation considerations for hallucination mitigation strategies in healthcare contexts emerged from our analysis. The reviewed studies addressed hallucination mitigation across various healthcare contexts, with clinical decision support systems emphasizing the need for high precision and recall of factual medical information, with strong preferences for RAG and human-in-the-loop approaches. Medical education applications focused on ensuring accurate representation of medical knowledge, often employing knowledge graph integration and specialized training approaches. Patient-facing applications prioritized avoiding harmful misinformation, frequently utilizing RAG with trusted source retrieval and human review. Medical documentation systems focused on faithful representation of patient information, commonly using RAG integrated with electronic health records [47].

Studies highlighted varying resource requirements across strategies. RAG and knowledge graph approaches typically required computational resources for real-time implementation, particularly for complex medical queries. The effectiveness of RAG and knowledge graph approaches depended heavily on the quality and comprehensiveness of the knowledge sources used. Human-in-the-loop approaches required ongoing access to medical expertise, raising questions about scalability and sustainability. Self-reflection frameworks and specialized training approaches often involved complex implementation but potentially fewer resources during deployment. Implementation considerations for red teaming included the need for structured testing methodologies with realistic healthcare scenarios. Structured frameworks, as proposed by Gadiko [11], typically involved multiphase processes: team assembly with diverse medical and technical expertise, development of testing frameworks with healthcare-specific scenarios, systematic execution of tests, and development of mitigation strategies based on findings.

Interoperability standards, particularly HL7 FHIR, were a recurrent implementation theme but were not consistently operationalized as explicit hallucination controls. Prior interoperability studies indicate that FHIR resource modeling, transport-level exchange constraints, and emergency-message pathways can be used as control points for provenance, validation, and schema-constrained generation [15–20]. In practice, this means linking hallucination mitigation to data contracts (e.g., FHIR resources and value sets), not only to prompt design.

Data-cleaning pipelines are necessary but insufficient as stand-alone hallucination controls. Multi-layer and ontology-driven cleaning methods improve consistency and compression of source data, but they do not by themselves prevent generative fabrication at inference time when models extrapolate beyond available evidence [48, 49]. Effective quality control therefore requires coupling upstream data-quality methods with downstream generation-time verification and monitoring.

Several studies discussed regulatory and ethical considerations for implementing hallucination mitigation strategies in healthcare, including transparency requirements (the need to clearly communicate the limitations of AI systems and the potential for hallucinations, even with mitigation strategies in place), responsibility frameworks (questions about liability and responsibility when hallucinations occur despite mitigation efforts), evaluation standards (the need for standardized evaluation frameworks to assess hallucination risks in healthcare AI systems), and monitoring requirements (recommendations for ongoing monitoring of deployed systems to detect and address novel forms of hallucinations).

To support practical implementation decisions, we provide an at-a-glance strategy tradeoff table, a context-to-approach selection table with suggested measures, and a limitations decision table in Appendix H.

## Discussion

### Summary of evidence

This systematic review identified seven primary categories of strategies for reducing hallucinations in AI systems designed for medical and healthcare applications, plus an additional category of user-facing communication strategies. The evidence suggests that effective hallucination mitigation in healthcare contexts requires multi-faceted approaches that combine technical solutions with domain expertise and appropriate communication strategies.

Retrieval-Augmented Generation (RAG) emerged as a particularly common approach, offering the ability to ground AI responses in verified medical knowledge without requiring extensive model retraining. The effectiveness of RAG approaches depended on the quality and comprehensiveness of the underlying retrieval system and knowledge sources, with healthcare-specific adaptations showing the strongest results.

Knowledge graph integration demonstrated substantial benefits, particularly for addressing fact-conflicting hallucinations by providing structured relationships between medical concepts. However, the development and maintenance of comprehensive medical knowledge graphs remained challenging, as noted by Agrawal et al. [26] and Penkov [28], due to the need for comprehensive and up-to-date medical knowledge graphs, computational overhead for large-scale implementations, and difficulties in handling medical information that falls outside the structured knowledge representation.

Self-reflection and verification frameworks, particularly those adapted for healthcare contexts like Chain-of-Medical-Thought (CoMT), showed promise by guiding models to follow clinically relevant reasoning patterns. Kim et al. [7] highlighted that these inference techniques can effectively reduce hallucination rates in medical AI applications, though their effectiveness varied depending on the complexity of the medical query. The Chain-of-Medical-Thought approach developed by Jiang et al. [30] demonstrated particular promise, reducing catastrophic hallucinations by 38% in medical report generation tasks.

The development of specialized benchmarks and evaluation metrics like MedHallBench, ACHMI, MediHall Score, and semantic entropy approaches represented important steps toward standardized assessment of hallucination mitigation strategies in healthcare contexts. These metrics provided more clinically relevant assessments by considering the varying severity and impact of different types of medical misinformation. The adoption of standardized categorization systems for hallucinations (catastrophic, critical, and attribute) to quantify the clinical significance of different types of errors.

Human-in-the-loop approaches consistently demonstrated effectiveness but faced challenges related to scalability and workflow integration. Finding the optimal balance between automation and human oversight remains an open question for healthcare applications, particularly as noted by Hatem et al. [12]

Specialized training techniques, particularly those leveraging reinforcement learning from expert feedback, showed promise but typically required substantial computational resources and expert involvement. The trade-off between hallucination reduction and maintaining model generative capabilities remains a challenge.

Communication-based strategies, including polite or cautious AI communication styles and transparent uncertainty expression, showed modest but meaningful effects on helping users detect and appropriately evaluate potential hallucinations. These strategies may offer complementary benefits when technical solutions cannot entirely eliminate hallucination risks.

Red teaming emerged as a valuable approach for identifying novel vulnerabilities that other methods might miss. Chang et al. [8] demonstrated that systematic red teaming can identify complex patterns of hallucination triggers in medical contexts. However, as Feffer et al. [45] highlighted, the effectiveness of red teaming depends heavily on team composition, methodology, and implementation considerations, with interdisciplinary teams showing the best results.

### Assumptions, lessons learned, and changes needed

Our synthesis relies on three practical assumptions: first, that retrieved knowledge sources and structured medical repositories are sufficiently current for clinical use; second, that healthcare organizations can maintain minimal expert oversight capacity for high-risk outputs; and third, that evaluation metrics used in the included studies are reasonable proxies for clinically meaningful safety risk. Across studies, three lessons were consistent. First, no single mitigation layer was adequate in isolation. Second, interoperability and data-governance architecture (e.g., schema constraints, provenance, and source traceability) materially influenced hallucination risk. Third, mitigation effectiveness depended strongly on implementation context, especially workflow burden and escalation pathways for uncertain outputs.

Based on these lessons, we identify three near-term changes for deployment programs: (1) require source attribution and uncertainty signaling in user-facing outputs, (2) align generation pipelines with structured interoperability standards such as HL7 FHIR where feasible, and (3) implement post-deployment surveillance with predefined triggers for model rollback or human escalation when hallucination thresholds are exceeded.

### Comparison with previous reviews

Our findings build upon and extend previous reviews in this area. Tonmoy et al. [4] surveyed hallucination mitigation techniques in LLMs more generally, identifying 32 techniques across different domains. Our review complements their work by focusing specifically on healthcare applications and providing a detailed analysis of implementation considerations in clinical contexts. Relative to that broader survey, we place stronger emphasis on clinical workflow constraints, domain-specific evaluation measures, and the role of human oversight in high-risk decisions; these differences are complementary rather than contradictory.

Ji et al. [10] provided a broad survey of hallucination in natural language generation, but not a healthcare-specific synthesis of implementation constraints. Our review extends that broader literature by identifying healthcare-specific adaptations and practical deployment considerations for each mitigation strategy category, including interoperability standards, clinical escalation pathways, and impact-weighted error framing.

Ahmad et al. [44] discussed creating trustworthy LLMs for healthcare with a section on hallucination mitigation but did not provide a comprehensive review of available strategies. Our systematic approach provides a comprehensive analysis of the available evidence and effectiveness of different approaches.

To contextualize adjacent interoperability and data-quality research requested by the reviewer, we added a focused comparison of related HIE/FHIR/data-cleaning studies and their relationship to hallucination control objectives (Appendix F, Table F.1) and a high-level functional mapping figure (Appendix F.2).

### Implications for practice and future outlook

Healthcare organizations considering the implementation of AI systems should prioritize those that incorporate robust hallucination mitigation strategies, particularly those that combine multiple approaches. Retrieval-augmented generation using high-quality medical knowledge sources appears to be a minimum requirement for responsible deployment.

The selection of specific mitigation strategies should be guided by the intended application context, available resources, and risk level. High-stakes applications like clinical decision support warrant more comprehensive approaches, potentially including human oversight, while lower-risk applications like general health information may be adequately served by RAG or knowledge graph approaches alone.

Human oversight remains essential, particularly for high-stakes healthcare applications. Organizations should develop clear protocols for human review of AI-generated content and establish feedback mechanisms to continuously improve system performance. Transparency about the limitations of AI systems is crucial for building trust and setting appropriate expectations among healthcare providers and patients.

A key finding from our review is that healthcare organizations should consider implementing multiple complementary hallucination mitigation strategies rather than relying on a single approach. The evidence suggests that combined approaches (e.g., RAG with knowledge graph validation, or self-reflection with human oversight) provide more robust protection against hallucinations than any individual strategy.

Several research priorities emerged from this review, including the development of standardized evaluation frameworks with metrics that reflect clinical significance rather than just statistical measures. While efforts like Med-HALT [34] and MediHall [33] represent important steps, wider adoption and validation of these frameworks across different healthcare domains is needed. Longitudinal studies in real-world clinical settings are needed to understand the real-world effectiveness of hallucination mitigation strategies and their impact on clinical workflows and outcomes.

Research into the optimal combination of mitigation strategies for different healthcare contexts could lead to more effective solutions. Few studies have systematically compared different combinations of strategies, representing a gap in the literature. Further research is needed on how to effectively integrate human expertise into AI workflows to minimize hallucinations while maximizing efficiency, including the development of user interfaces that facilitate human review and correction of AI-generated content.

More work is needed on adapting general hallucination mitigation strategies to the specific requirements of different medical specialties and contexts. For example, radiology, psychiatry, and emergency medicine may each benefit from tailored approaches. Developing techniques that not only identify potential hallucinations but also explain the reasoning could enhance trust and usability, aligning with broader calls for explainable AI in healthcare [50].

Beyond direct clinical care, these findings are applicable to adjacent sectors that use high-stakes health information workflows, including public health reporting, payer utilization management, emergency response coordination, clinical trial screening, and digital health triage services. In each case, the same core design principle applies: pair domain-grounded retrieval or structured constraints with verification and escalation logic proportional to downstream risk.

The rapid pace of development in AI technology, particularly LLMs, means that hallucination mitigation strategies will need to evolve continually. Research efforts should focus not only on developing new mitigation approaches but also on ensuring that existing strategies remain effective as AI capabilities advance.

### Limitations

This systematic review has several limitations. First, the rapidly evolving nature of the field means that new approaches may have emerged since the completion of our search. Second, the heterogeneity of evaluation methodologies made direct comparisons between studies challenging. Third, many studies were conducted by AI developers rather than healthcare professionals, potentially limiting clinical relevance.

Additionally, our review was constrained by the limitations of the primary studies themselves. Many studies evaluated hallucination mitigation strategies in controlled environments rather than real-world clinical settings, raising questions about their generalizability. The lack of standardized evaluation metrics also made it difficult to draw definitive conclusions about relative effectiveness.

Finally, publication bias may have influenced our findings, as studies demonstrating positive results for hallucination mitigation are more likely to be published than those showing limited or negative effects. The predominance of recent publications also suggests that the field is still in its early stages, with many of the proposed strategies yet to undergo rigorous long-term evaluation.

Related research attempts also face recurring operational challenges, including reliance on synthetic prompts, single-center datasets, and limited prospective testing under real workload constraints. These limitations reduce certainty for deployment decisions but still provide important value as early-stage design evidence and hypothesis-generating work for larger pragmatic evaluations.

To make these limits actionable for implementers, we summarize what is currently known and what questions should be answered before deployment in a dedicated limitations decision table (Appendix H.3).

## Conclusions

Hallucinations represent an important barrier to the safe and effective deployment of AI systems in healthcare settings. This systematic review identified seven primary categories of strategies for reducing hallucinations in medical AI applications, with the most promising approaches combining multiple strategies, particularly those integrating domain-specific knowledge retrieval with verification mechanisms and adversarial testing.

Retrieval-Augmented Generation, when implemented with high-quality medical knowledge sources, demonstrated the most consistent improvements in reducing factual errors. Knowledge graph integration provided a structured framework for maintaining factual consistency, particularly for complex medical relationships. Self-reflection frameworks showed promise for guiding models through clinical reasoning patterns, while specialized benchmarks and metrics offered more clinically relevant evaluation approaches. Human-in-the-loop systems provided the highest accuracy but faced scalability challenges, specialized training techniques demonstrated variable effectiveness depending on implementation, and red teaming approaches were valuable for identifying novel vulnerabilities that other methods might miss.

While progress has been made in developing these strategies, rigorous evaluation in real-world clinical settings remains limited. Future research should focus on developing standardized evaluation frameworks, investigating the effectiveness of combined approaches, and optimizing the integration of human oversight into AI workflows.

As healthcare organizations increasingly explore the potential of AI applications, implementing robust hallucination mitigation strategies will be essential for realizing the benefits of these technologies while minimizing potential risks to patient care. The field would benefit from greater collaboration between AI researchers, healthcare professionals, and regulatory bodies to establish standards and best practices for hallucination mitigation in medical AI systems.

Ultimately, the goal should be to develop AI systems that not only minimize hallucinations but also clearly communicate their limitations and uncertainties, enabling healthcare professionals to use these tools appropriately and effectively in clinical practice. This will require ongoing research, evaluation, and refinement of hallucination mitigation strategies as AI technology continues to evolve.

The primary beneficiaries of this work are clinicians, patients, health-system leaders, developers, informatics teams, educators, and regulators. Clinicians and patients benefit through safer decision support and clearer uncertainty signaling; developers and informatics teams benefit from an implementation typology tied to interoperability and workflow constraints; regulators and administrators benefit from clearer targets for evaluation and monitoring requirements.

Our dissemination approach is to pair this review with actionable implementation artifacts (workflow diagram, comparison tables, and extraction framework in the Appendix) and to communicate findings across clinical informatics, health services research, and AI safety venues.

## Electronic supplementary material

Below is the link to the electronic supplementary material.

Supplementary Material 1

## Data Availability

Aggregate data used to generate reported counts and consistency checks are provided in the supplementary appendix (Appendix C-D).
