# Supplementary material for: Mitigating hallucinations in healthcare AI: a systematic review of evidence-based strategies
Source: BMC Health Serv Res. 2026 Jun 6;26:1115. doi: 10.1186/s12913-026-14851-1 (PMC13470931; doi:10.1186/s12913-026-14851-1)
Supplement: Supplementary file 1 [file 12913_2026_14851_MOESM1_ESM.docx]

# Supplementary Appendix

**Mitigating Hallucinations in Healthcare AI: A Systematic Review of Evidence-Based Strategies**

## Appendix A: PRISMA 2020 Compliance Summary

| **PRISMA item** | **What we report** | **Location in revised manuscript** |
| --- | --- | --- |
| Title/Abstract | Systematic review title and structured abstract | Main text title page; Abstract |
| Rationale/Objectives | Clinical rationale, healthcare-specific focus, explicit objectives | Introduction |
| Information sources and search strategy | Databases, date limits, and full query logic | Methods; Appendix B |
| Selection process | Staged screening and consensus-based eligibility assessment | Methods |
| Data extraction and quality appraisal | Standardized extraction and adapted JBI appraisal | Methods; Appendix E |
| Results of selection | Record counts through screening and inclusion | Results; Figure 1; Appendix C |
| Study characteristics and synthesis | Aggregate characteristics and strategy categories | Results; Table 1; Table 2; Appendix D |
| Limitations and implications | Methodological limits, assumptions, implementation implications | Discussion; Conclusions |
| Data/materials availability | Supplementary aggregate data and consistency checks | Declarations; Appendix C-D |

## Appendix B: Detailed Search Strategy

### B.1 PubMed

(("artificial intelligence"[MeSH Terms] OR "artificial intelligence"[Title/Abstract] OR "AI"[Title/Abstract] OR "large language model*"[Title/Abstract] OR "LLM*"[Title/Abstract] OR "generative AI"[Title/Abstract] OR "ChatGPT"[Title/Abstract] OR "generative pre-trained transformer"[Title/Abstract] OR "transformer model*"[Title/Abstract] OR "foundation model*"[Title/Abstract]) AND ("hallucinat*"[Title/Abstract] OR "factual error*"[Title/Abstract] OR "misinformation"[Title/Abstract] OR "reliability"[Title/Abstract] OR "accuracy"[Title/Abstract] OR "truthfulness"[Title/Abstract] OR "fabricat*"[Title/Abstract]) AND ("medical"[Title/Abstract] OR "healthcare"[Title/Abstract] OR "clinical"[Title/Abstract] OR "diagnosis"[Title/Abstract] OR "health"[Title/Abstract] OR "patient"[Title/Abstract] OR "physician"[Title/Abstract] OR "doctor"[Title/Abstract] OR "hospital"[Title/Abstract] OR "medical education"[Title/Abstract] OR "Medicine"[MeSH Terms] OR "Healthcare"[MeSH Terms])) AND ("2019/01/01"[Date - Publication] : "2025/04/30"[Date - Publication])

### B.2 IEEE Xplore

(("Document Title":"artificial intelligence" OR "Document Title":"AI" OR "Document Title":"large language model*" OR "Document Title":"LLM*" OR "Document Title":"generative AI" OR "Document Title":"ChatGPT" OR "Document Title":"transformer model*" OR "Document Title":"foundation model*") OR ("Abstract":"artificial intelligence" OR "Abstract":"AI" OR "Abstract":"large language model*" OR "Abstract":"LLM*" OR "Abstract":"generative AI" OR "Abstract":"ChatGPT" OR "Abstract":"transformer model*" OR "Abstract":"foundation model*")) AND (("Document Title":"hallucinat*" OR "Document Title":"factual error*" OR "Document Title":"misinformation" OR "Document Title":"reliability" OR "Document Title":"accuracy" OR "Document Title":"truthfulness" OR "Document Title":"fabricat*") OR ("Abstract":"hallucinat*" OR "Abstract":"factual error*" OR "Abstract":"misinformation" OR "Abstract":"reliability" OR "Abstract":"accuracy" OR "Abstract":"truthfulness" OR "Abstract":"fabricat*")) AND (("Document Title":"medical" OR "Document Title":"healthcare" OR "Document Title":"clinical" OR "Document Title":"diagnosis" OR "Document Title":"health" OR "Document Title":"patient") OR ("Abstract":"medical" OR "Abstract":"healthcare" OR "Abstract":"clinical" OR "Abstract":"diagnosis" OR "Abstract":"health" OR "Abstract":"patient"))

Filters: 2019-2025.

### B.3 ACM Digital Library

[[All: "artificial intelligence"] OR [All: "large language model*"] OR [All: "LLM*"] OR [All: "generative AI"] OR [All: "ChatGPT"] OR [All: "transformer model*"] OR [All: "foundation model*"]] AND [[All: "hallucinat*"] OR [All: "factual error*"] OR [All: "misinformation"] OR [All: "reliability"] OR [All: "accuracy"] OR [All: "truthfulness"] OR [All: "fabricat*"]] AND [[All: "medical"] OR [All: "healthcare"] OR [All: "clinical"] OR [All: "diagnosis"] OR [All: "health"] OR [All: "patient"] OR [All: "physician"] OR [All: "doctor"] OR [All: "hospital"]]

Additional filter: published since 2019.

### B.4 Scopus

TITLE-ABS-KEY(("artificial intelligence" OR "AI" OR "large language model*" OR "LLM*" OR "generative AI" OR "ChatGPT" OR "transformer model*" OR "foundation model*") AND ("hallucinat*" OR "factual error*" OR "misinformation" OR "reliability" OR "accuracy" OR "truthfulness" OR "fabricat*") AND ("medical" OR "healthcare" OR "clinical" OR "diagnosis" OR "health" OR "patient" OR "physician" OR "doctor" OR "hospital")) AND PUBYEAR > 2018 AND PUBYEAR < 2026

### B.5 Web of Science

TS=(("artificial intelligence" OR "AI" OR "large language model*" OR "LLM*" OR "generative AI" OR "ChatGPT" OR "transformer model*" OR "foundation model*") AND ("hallucinat*" OR "factual error*" OR "misinformation" OR "reliability" OR "accuracy" OR "truthfulness" OR "fabricat*") AND ("medical" OR "healthcare" OR "clinical" OR "diagnosis" OR "health" OR "patient" OR "physician" OR "doctor" OR "hospital"))

Timespan: 2019-2025.

### B.6 arXiv

"artificial intelligence" OR "large language model" OR "LLM" OR "generative AI" OR "ChatGPT" OR "transformer model" OR "foundation model" AND "hallucination" OR "factual error" OR "misinformation" OR "reliability" OR "accuracy" OR "truthfulness" OR "fabrication" AND "medical" OR "healthcare" OR "clinical" OR "diagnosis" OR "health" OR "patient" OR "physician" OR "doctor" OR "hospital"

Filters: From 2019-01-01; categories cs.AI, cs.CL, cs.LG, stat.ML.

## Appendix C: Screening Flow and Reproducibility Checks

### C.1 PRISMA flow counts used in the manuscript

| **Stage** | **Count** |
| --- | --- |
| Records identified | 427 |
| Duplicates removed | 71 |
| Records screened (title/abstract) | 356 |
| Full-text assessed | 91 |
| Full-text excluded | 47 |
| Included studies | 44 |
| Excluded: no healthcare-specific context | 22 |
| Excluded: hallucinations in human patients | 14 |
| Excluded: insufficient methodological detail | 11 |

### C.2 Deterministic consistency checks

- 427 - 71 = 356
- 91 - 47 = 44
- 22 + 14 + 11 = 47

These values are reported directly here so the arithmetic can be independently verified.

## Appendix D: Aggregate Study Data Used for Results

### D.1 Study characteristics (aggregate counts)

| **Category** | **Type** | **Count** |
| --- | --- | --- |
| Publication type | Original research articles | 30 |
| Publication type | Systematic reviews | 7 |
| Publication type | Technical papers | 7 |
| Publication date | 2019-2022 | 7 |
| Publication date | 2023-2025 | 37 |
| Publication venue | Medical informatics journals | 17 |
| Publication venue | AI/computer science conferences | 14 |
| Publication venue | AI preprint repositories | 9 |
| Publication venue | General medical journals | 4 |
| Geographical distribution | North America | 20 |
| Geographical distribution | Europe | 12 |
| Geographical distribution | Asia | 12 |
| Healthcare domain | General medicine | 15 |
| Healthcare domain | Radiology/medical imaging | 11 |
| Healthcare domain | Clinical decision support | 8 |
| Healthcare domain | Medical education | 5 |
| Healthcare domain | Other specialized areas | 5 |
| Quality assessment | High quality | 11 |
| Quality assessment | Moderate quality | 26 |
| Quality assessment | Low quality | 7 |

These category totals are reported directly here for independent verification.

### D.2 Strategy frequency counts (non-mutually exclusive)

| **Strategy class** | **Study count** |
| --- | --- |
| Retrieval-Augmented Generation | 18 |
| Knowledge graph integration | 12 |
| Self-reflection and verification frameworks | 10 |
| Specialized benchmarks and evaluation metrics | 8 |
| Human-in-the-loop approaches | 7 |
| Specialized training techniques | 10 |
| User-facing communication strategies | 5 |
| Red teaming | 7 |

These strategy frequencies are reported directly here for independent verification.

### D.3 Reproducibility equations

The following equations are sufficient to reproduce all aggregate consistency checks:

- 427 - 71 = 356
- 91 - 47 = 44
- 22 + 14 + 11 = 47
- Publication type subtotal: 30 + 7 + 7 = 44
- Publication date subtotal: 7 + 37 = 44
- Venue subtotal: 17 + 14 + 9 + 4 = 44
- Region subtotal: 20 + 12 + 12 = 44
- Domain subtotal: 15 + 11 + 8 + 5 + 5 = 44
- Quality subtotal: 11 + 26 + 7 = 44

### D.4 Numeric-claim provenance and non-synthetic data statement

All quantitative values reported in the revised manuscript follow one of three provenance classes:

1. Direct study counts from the screening and extraction workflow (Appendix C-D.2).
2. Direct percentages/effect sizes reported by included primary studies and cited in text.
3. Deterministic percentages computed from direct counts (for example, quality proportions in Figure 2 caption).

No simulated, placeholder, imputed, or synthetic quantitative values were introduced in this revision.

Reproducible derived percentages used in the text:

- Moderate quality studies: 26 / 44 = 59.1% (reported as 59%).
- High-quality studies: 11 / 44 = 25.0% (reported as 25%).

## Appendix E: Quality Assessment Tool (Instrument)

| **Quality criterion** | **Yes** | **No** | **Unclear** | **Not applicable** |
| --- | --- | --- | --- | --- |
| 1. Research objective clearly stated |  |  |  |  |
| 2. AI system/model adequately described |  |  |  |  |
| 3. Mitigation strategy clearly defined |  |  |  |  |
| 4. Healthcare implementation context specified |  |  |  |  |
| 5. Evaluation methodology appropriate |  |  |  |  |
| 6. Evaluation metrics appropriate and defined |  |  |  |  |
| 7. Sample/dataset adequately described |  |  |  |  |
| 8. Comparison/control included |  |  |  |  |
| 9. Results reported in sufficient detail |  |  |  |  |
| 10. Study limitations discussed |  |  |  |  |
| 11. Clinical implications discussed |  |  |  |  |
| 12. Ethical considerations addressed |  |  |  |  |

Scoring rule used in the manuscript:

- Low quality: 4 or fewer "Yes"
- Moderate quality: 5-8 "Yes"
- High quality: 9 or more "Yes"

## Appendix F: Related Interoperability and Data-Quality Work

### F.1 Comparison table for related HIE/FHIR/data-quality studies

| **Related study** | **Primary contribution** | **Value for healthcare AI quality** | **Why not sufficient alone for hallucination control** |
| --- | --- | --- | --- |
| Kiourtis et al., 2021 (Bluetooth-based HIE protocol) | Protocol for short-range exchange of health information | Supports resilient message transport in constrained settings | Transport reliability does not guarantee factual correctness of generated text |
| Vidakis et al., 2020 (short-range wireless comparative study) | Performance comparison across short-range communication options for health information exchange | Informs infrastructure choices for data movement | Network/channel quality does not address model fabrication or reasoning errors |
| Symvoulidis et al., 2021 (medical emergency HIE) | Emergency-focused data exchange workflow | Demonstrates urgency-sensitive data transfer needs | Emergency exchange speed can coexist with model-level hallucination risk |
| Kiourtis et al., 2023 (HL7 FHIR health determinants categorization) | Structuring determinants into FHIR-compatible paradigms | Enables schema-constrained data representation and provenance | Structured inputs reduce ambiguity but cannot prevent unsupported generation |
| Kouremenou et al., 2024 (interoperable data modeling process) | Process-level approach to interoperability modeling | Offers repeatable interoperability design logic | Interoperability design alone does not evaluate output factuality |
| Mavrogiorgou et al., 2019 (IoMT -> HL7 FHIR via 5G slicing) | Pipeline from IoMT acquisition to FHIR transformation | Highlights real-time transformation and integration feasibility | Real-time ingestion can still propagate or amplify hallucinated narratives |
| Mavrogiorgos et al., 2022 (multi-layer data cleaning) | Multi-layer approach for healthcare data cleaning | Improves consistency and reduces upstream data defects | Clean source data does not eliminate generative extrapolation errors |
| Kiourtis et al., 2022 (ontology-driven cleaning/compression) | Ontology-guided cleaning with lossless compression goals | Strengthens semantic consistency in source datasets | Ontology consistency does not enforce inference-time factual grounding |

### F.2 High-level functionality map

```
[Clinical data sources: EHR, HIE feeds, literature]

                  |

                  v

[Interoperability layer: exchange and emergency messaging]

                  |

                  v

[FHIR/data-model layer: resource mapping, value-set constraints, provenance]

                  |

                  v

[LLM hallucination controls: RAG, KG validation, self-verification]

             /                         \\

            v                           v

[Human oversight]            [Monitoring and red teaming]

                                       |

                                       v

                         [Feedback to source/control design]
```

## Appendix G: Replication Workflow Diagram (stepwise)

```
[Start]

   |

   v

[1. Define objective and scope]

   |

   v

[2. Pre-specify protocol and search terms]

   |

   v

[3. Search six databases]

   |

   v

[4. De-duplicate records]

   |

   v

[5. Title/abstract screening]

   |

   v

<Eligible for full text?>

   | Yes                           | No

   v                               v

[6. Full-text eligibility]   [Exclude with reason]

   |

   v

[7. Structured data extraction]

   |

   v

[8. Adapted JBI quality appraisal]

   |

   v

[9. Narrative synthesis by strategy category]

   |

   v

[End: comparative findings, implementation guidance, and gaps]
```

This stepwise flow diagram provides the concrete step order and decision gate used to replicate the review workflow described in Methods.

## Appendix H: Strategy Selection Aids for Developers and Implementers

### H.1 At-a-glance strategy tradeoff table (7 core strategies)

| **Strategy** | **What it can do well** | **What it cannot do alone** | **Typical advantages** | **Typical disadvantages** | **Example implementation pattern** |
| --- | --- | --- | --- | --- | --- |
| Retrieval-Augmented Generation (RAG) | Grounds responses in retrieved evidence and reduces unsupported claims | Cannot guarantee correctness if retrieval corpus is incomplete or low quality | Updatable without full model retraining; strong factuality gains | Retrieval latency; source-quality dependence | Query -> retrieve guideline passages -> answer with citations |
| Knowledge Graph Integration | Enforces structured medical relationships and consistency constraints | Cannot cover concepts absent from the graph; may miss nuanced narrative context | Strong for relation-heavy reasoning | Graph maintenance overhead; ontology drift risk | Map entities to KG -> validate generated relations before output |
| Self-reflection / verification loops | Detects internal contradictions and prompts model self-correction | Can propagate earlier errors across loop iterations | Improves consistency without external reviewers for every output | Extra compute and latency | Draft answer -> verification pass -> corrected answer with uncertainty |
| Specialized benchmarks and metrics | Quantifies hallucination severity and supports model comparison | Does not itself mitigate errors at inference time | Enables governance and monitoring thresholds | Measurement heterogeneity across domains | Run Med-HALT/MediHall-style evaluations pre-deployment |
| Human-in-the-loop (HITL) | Catches high-risk hallucinations before use in care pathways | Cannot fully scale across all low-risk outputs | Strongest safety net for high-stakes tasks | Workflow burden; reviewer disagreement | Auto-flag uncertain outputs -> clinician adjudication |
| Specialized training techniques | Reduces baseline hallucination tendency through model adaptation | Cannot fully prevent out-of-distribution failures | Can improve base model robustness | Requires curated data and compute | Contrastive/uncertainty-aware fine-tuning on clinical corpora |
| Red teaming | Finds failure modes that standard tests miss | Does not by itself provide production-time controls | Surfaces adversarial vulnerabilities early | Resource-intensive; team composition sensitive | Multidisciplinary adversarial prompt campaigns + mitigation loop |

### H.2 Application-oriented strategy/measure mapping table

| **Primary use case** | **Recommended strategy bundle** | **Hallucination measures most useful for this context** | **Why this bundle is preferred** |
| --- | --- | --- | --- |
| Clinical decision support (high-risk) | RAG + KG validation + HITL + red teaming | Severe-error weighted metrics (e.g., catastrophic/critical classes), expert disagreement rate, escalation rate | Balances factual grounding with explicit clinical safety gates |
| Patient-facing Q&A and triage | RAG + uncertainty communication + targeted HITL for flagged cases | Unsafe-advice rate, unsupported-claim rate, user-calibrated trust measures | Reduces misinformation while preserving usability |
| Medical documentation/summarization | RAG + self-verification + source citation checks | Omission/fabrication rates, citation-faithfulness, factual consistency scores | Improves traceability and post-hoc auditability |
| Medical education tools | KG integration + RAG + benchmark-driven monitoring | Concept-level factuality, relation-consistency errors, benchmark pass rates | Supports stable teaching content and conceptual coherence |
| Emergency and interoperable exchange settings | Interoperability constraints (FHIR/schema) + RAG + red teaming | Latency-to-safe-response, schema-conformance errors, hallucination-under-stress tests | Maintains safety under time pressure and heterogeneous data flows |

### H.3 Limitations decision table (knowns vs pre-deployment questions)

| **What is currently known** | **What remains uncertain / should be asked before deployment** |
| --- | --- |
| Combined strategies generally outperform single methods in healthcare contexts | Which combination is optimal for a specific specialty/workflow and acceptable latency budget? |
| HITL improves end-stage safety, especially for high-risk tasks | What adjudication process is used when experts disagree, and what is the escalation rule? |
| RAG and KG methods improve factual grounding when data quality is high | Are retrieval sources complete, current, and locally relevant for intended users? |
| Specialized metrics can detect clinically meaningful error classes | Which metrics map to real patient-risk thresholds in the target deployment? |
| Red teaming identifies previously unseen vulnerabilities | How often will red teaming be repeated post-deployment and who owns remediation timelines? |
| Data-cleaning/interoperability controls improve upstream consistency | How will inference-time fabrication be monitored and corrected in production? |
